# ELDER ABUSE BY FAMILY CAREGIVER: DOES COGNITIVE IMPAIRMENT REALLY MATTER?

**DOI:** 10.1093/geroni/igz038.1685

**Published:** 2019-11-08

**Authors:** Elsie Yan

**Affiliations:** The Hong Kong Polytechnic University, Hong Kong, Hong Kong

## Abstract

Previous research suggested that cognitive impairment is a risk factor for elder abuse. Persons with dementia experience elevated risk of abuse as compared to the general aging population. The present study compared rates of abuse reported by family caregivers of older persons with and without dementia. A total of 693 family caregivers participated, among which 592 were providing care older persons with dementia and 101 were providing care to older persons without. Participants provided information on their demographic characteristics, care recipient physical functioning (Instrumental Activities of Daily Living), behavioral problems (Cohen Mansfield Agitation Inventory), caregiver stress (Zarit Burden Interview), emotional and instrumental social support, and abusive behaviors directed at the care recipients (Conflict Tactics Scale and Potentially Harmful Behaviors). Abuse is common in this sample: 46.8% reported potentially harmful behaviors, 52.7% reported psychological aggression, 11% physical assault, and 1.3% injury. No significant difference was observed between caregivers providing care to older persons with or without dementia (p>.05). A series of logistic regression was conducted to determine factors associated with abuse. Care recipient behavioral problems and caregiver burden were two prominent factors associated with potentially harmful behaviors and all forms of abuse. Behavioral problems are common in persons with cognitive impairment and many caregivers feel stressful managing them. It is plausible that cognitive impairments per sec do not increases risk of abuse, but the associated characteristics do. Helping family caregivers manage the caregiving situation and their expectation, positive appraisal and cognitive restructuring may help prevent elder abuse.

